# Atrophy of Optic Nerve in Erysipelas of the Face

**Published:** 1879-12

**Authors:** Lucien Howe


### ATROPHY OF THE OPTIC NERVE IN ERYSIPELAS OF THE FACE—BY PARINAUD.

TRANSLATED FROM THE FRENCH BY LUCIEN HOWE, M. D.

An erysipelas of the face may be followed by impaired vision which sometimes shows itself only in one eye, and occasionally affects both simultaneously. In either event, after a longer or shorter time, an atrophic degeneration of the optic disk becomes apparent.

The writer has observed such a result in two cases, and has collected six others from medical literature, which leave no doubt as to their relation with the erysipelas. After detailing his own observations, he gives an analysis of the other cases, seeking by their comparison the cause and signification of this grave complication. From that it appears that the impaired vision which results under these circumstances is accompanied or followed by atrophy of the optic nerve. The neuritis (acute stage) has not, however, been recognized in any instance.—(*Arch. Gener. de Medicine.*)—*Annales d'Oculistique.*

